# Evaluating Dental Arch Relationships in Indonesian Patients with Operated Bilateral Cleft Lip and Palate Using Modified Huddart/Bodenham Index and Bauru-bilateral cleft lip and palate (BCLP) Yardstick

**DOI:** 10.4317/jced.61121

**Published:** 2024-03-01

**Authors:** Dwi Ariawan, Muhammad-Arfah Rachman, Nur Aini, Muhammad-Syafrudin Hak, Vera Julia, Lilies-Dwi Sulistyani, Norifumi Nakamura

**Affiliations:** 1 Department of Oral and Maxillofacial Surgery, Faculty of Dentistry, Universitas Indonesia, Jakarta, Indonesia; 2 Cleft Center, Harapan Kita Children and Mother Hospital, Jakarta, Indonesia

## Abstract

**Background:**

Patients with complete bilateral cleft lip and palate (CBCLP) have the most complex orofacial abnormalities despite its lowest incidence among cleft lip and palate (CLP) types. Impaired maxillary growth can result from surgical procedures in patients with CBCLP. This study evaluates dental arch relationships in Indonesian patients with CBCLP after undergoing CLP repair at Harapan Kita Children and Mother Hospital in Jakarta.

**Material and Methods:**

Using the modified Huddart and Bodenham (MHB) index and Bauru bilateral cleft lip and palate (BCLP) yardstick, three examiners assessed 17 study models in the 9-year age group and 13 study models in the 12-year age group, as well as two intraoral clinical photographs of two patients with CBCLP. The assessments were repeated three times within two weeks of each assessment time.

**Results:**

Patients with operated CBCLP (aged 9 and 12 years) had edge-to-edge tooth relations, which were categorized as a mild crossbite or mild deviation, and only required orthodontic treatment according to the two specified indicators.

**Conclusions:**

The CLP repair protocol used at the Harapan Kita Hospital effectively manages CBCLP cases with satisfactory results, suggesting the effectiveness of the MHB index and Bauru-BCLP yardstick in assessing dental arch relationships in patients with operated CBCLP.

** Key words:**Bilateral, cleft lip and palate, dental arch relationship, lip repair, palate repair.

## Introduction

Cleft lip and palate (CLP) are the most common congenital abnormality in the head and neck region. Globally, these abnormalities occur in approximately 1 in every 700 births, varying widely across geographic areas or ethnic groups (1-4). CLP etiology is multifactorial, with genetic impacts and variable interactions from environmental factors (2,3,5).

CLP-induced problems in children include disorders of jaw development and disproportion of the midface. Wound contraction and scar tissue formation after cleft surgery affect the maxillary growth and dentofacial structures of patients with CLP (6-8). The outcome of cleft surgery occurs either in an open bite or crossbite and can cause speech and mastication problems, reducing the quality of life in children with this condition (6-9).

The dental arch relationship (DAR), a crucial indicator of maxillofacial growth, is an essential part for assessing CLP treatment (6,9-11). Clinical tools for evaluating DARs abound, with the Goslon yardstick assuming precedence for unilateral cleft lip and palate cases. This yardstick has a proven sensitivity in the spatial discrepancy assessment between the maxillary and mandibular dental arches. This instrument categorizes DAR into five categories that reflect cleft treatment outcomes (10,11).

Particularly for bilateral cleft lip and palate (BCLP) cases, the Bauru-BCLP yardstick is a reliable measure for DAR evaluation in patients with this condition (9,12-14). This yardstick categorizes DAR into five grades, from the good arch form category (Grade 1) to the very poor arch form category (Grade 5), which indicates the need for orthognathic surgery. Scores 1 and 2 in the 12-year group could be combined in cases where intermediate orthodontic treatment improved some patients’ occlusal conditions (9,12-14).

In addition to the Bauru-BCLP yardstick, the modified Huddart and Bodenham (MHB) index is often utilized to assess DARs for BCLP cases. This system was originally used to assess unilateral cleft in patients by considering the buccopalatal relationship based on the frequency and severity of anterior and buccal crossbites to assess maxillary arch constriction in the primary dentition period (15-20). The main advantages of the MHB index are its ease of use, which requires no calibration, its adaptability in assessing various types of CLP, and its numerical score that favors statistical inference (17). Heidbuchel and Kuijpers-Jagtman (1997) then modified the MHB index by adding more scoring categories for the buccal segment, the canines, and molars (20,21). Tothill and Mossey (2007) reported using the MHB index to assess dental arch constriction in BCLP patients. The results showed that the MHB index is an objective, sensitive, and versatile tool for evaluating dental arch constriction in all cleft types, including BCLP (6). Bartzela *et al*. (2011) used the MHB index to assess BCLP cases and revealed that this index can categorize cleft treatment outcomes into categories similar to the Bauru-BCLP yardstick (14). The MHB index determines the existence of an anterior–posterior crossbite and this crossbite severity. Except for the lateral incisors, each maxillary tooth is scored using this index concerning its opposing tooth in the mandible. The lower overall score indicates a more constricted maxillary arch (14,21-23,). Estacio *et al*. (2023) assessed DAR in 96 complete unilateral cleft lip and palate patients having a two-stage palatoplasty using the MHB index. The MHB index is reported to be more representative of the severity caused by primary surgical treatment, which is easy to use, and the results have a high level of agreement for trained and untrained professionals (24).

Cleft Center at the Children and Mother Harapan Kita Hospital, Jakarta, is an Indonesia’s leading cleft center. Since its establishment in 1995, this hospital has performed more than 3,000 surgeries, with an average of 140-150 surgeries yearly. The care provided for cleft patients is integrated and comprehensive according to the Cleft Center treatment protocol and involves experts from various disciplines.

**Figure 1:**
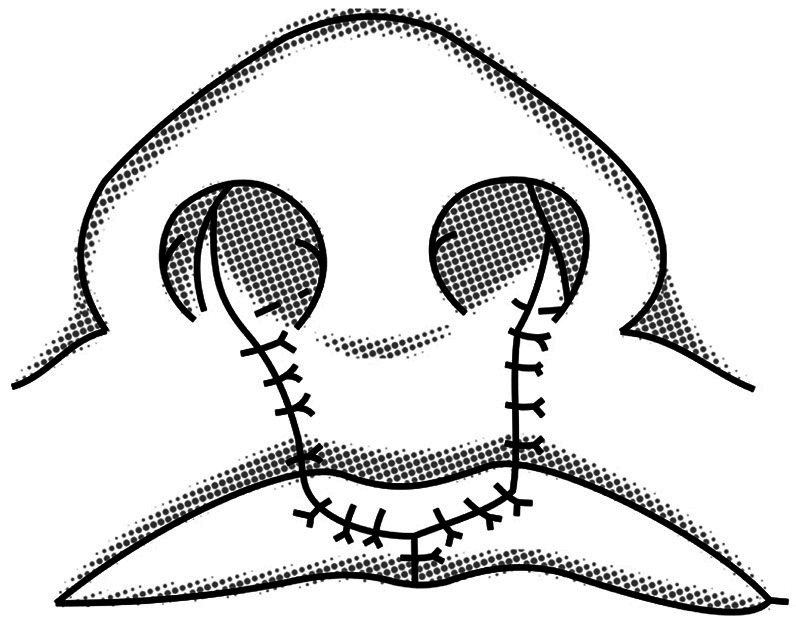
The modified Manchester lip repair.

**Figure 2:**
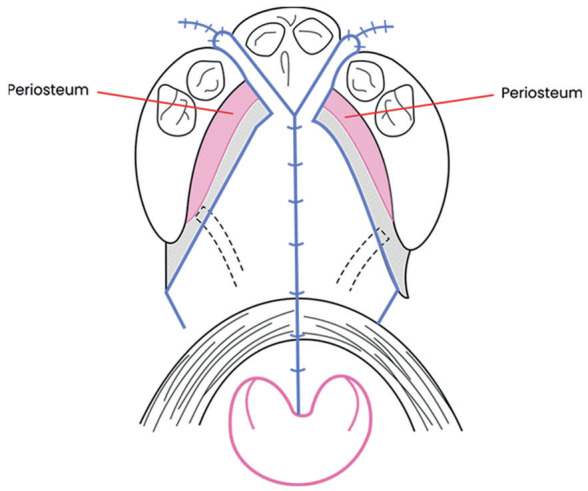
Cleft palate repair with a partial split flap technique.

Regarding complete bilateral cleft lip and palate (CBCLP) cases, two consultant oral and maxillofacial surgeons performed surgical management with a consistent protocol of lip repair using a modified Manchester technique (Fig. 1) and a protocol of palate repair using a modified pushback technique (Fig. 2) (25-27). The modified pushback palate repair was performed with a partial split lateral periosteal incision to preserve the periosteum at the anterior and lateral positions of the hard palate. Cleft palate repair using a partial split flap technique in which less denudation of the palatal bone exists can result in better transverse development of the maxillary dental arch compared to the full-thickness flap technique (Fig. 2) (27-30).

Studies assessing DARs after surgery in BCLP cases in Indonesia still need to be improved. This study evaluates DARs in Indonesian patients with BCLP using the MHB index and Bauru-BCLP yardstick, research conducted at Harapan Kita Hospital Jakarta.

## Material and Methods

This cross-sectional study employed Indonesian patients with CBCLP in the 9-year and 12-year age groups. The 9-year-old and 12-year-old yardsticks were chosen to represent the early mixed dentition and permanent dentition periods. The 9-year age group in this study were research subjects with an age range of 9+1 years (2920 days – 3650 days) when the dental impressions were taken. The 12-year age group in this study were research subjects aged 12+1 years (4015 days – 4745 days) when the dental impressions were taken. The patients underwent lip repair using the modified Manchester technique (Fig. 1) and palate repair using the modified pushback technique with a partial split lateral incision (Fig. 2) according to the treatment protocol at the specified hospital (Table 1). The study subjects comprised 32 patients born with BCLP, 19 patients in the age group of 9 years, and 13 patients in the age group of 12 years (Table 2). None had undergone orthodontic treatment.

**Table 1:**
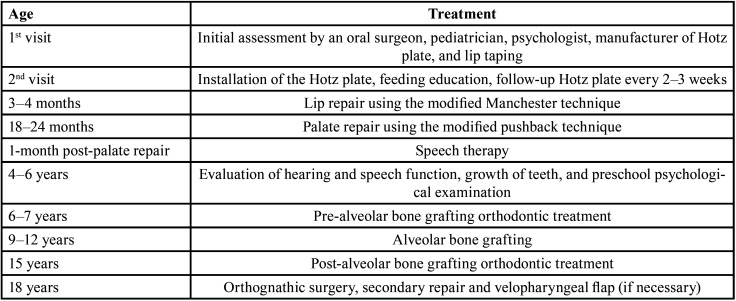
Treatment Protocol of the Cleft Lip and Palate Center, Harapan Kita Children and Mother Hospital.

**Table 2:**
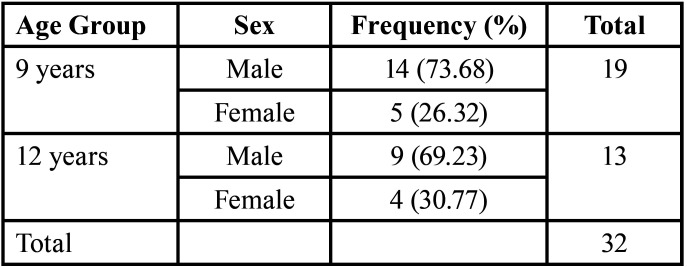
Distribution of 9- and 12-Year Age Group Subjects.

The study models for the 9-year and 12-year age groups were assessed using the MHB index and Bauru-BCLP yardstick. The assessment was performed by three examiners (DA, NA, MAR) who have been calibrated before. Measurements were repeated three times with an interval of 2 weeks to reduce data diversity, increase precision, and obtain correct and objective measurement data, especially if the two previous measurements (Duplo) showed significantly different results. While reliability was validated using the statistical value of Cronbach’s alpha, agreement between the two assessment instruments was validated using the Kappa statistical test.

**Figure 3:**
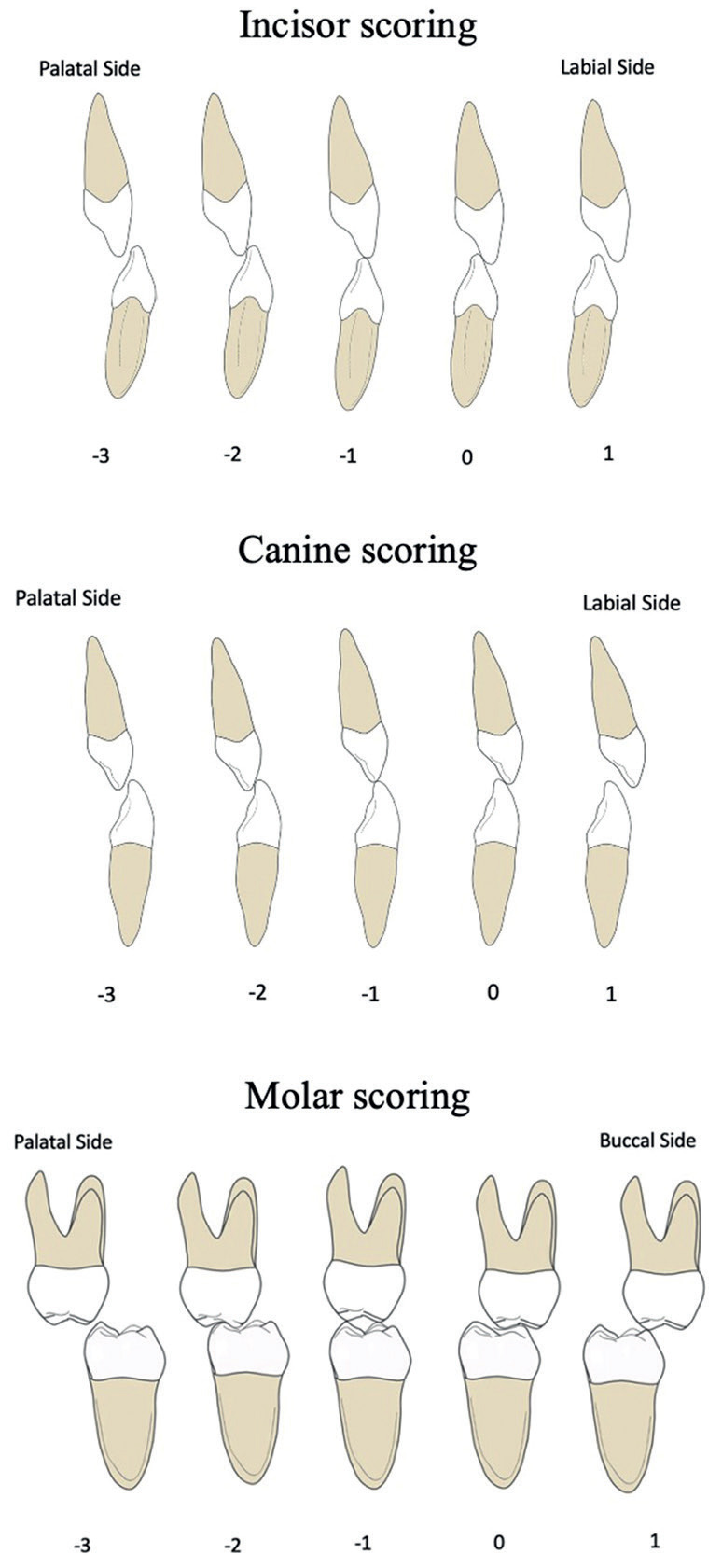
The modified Huddart-Bodenham (MHB) index for the scoring of buccopalatal relationships (modified from Heidbüchel and Kuijpers-Jagtman, 1997).

MHB assessment of DAR was based on the buccopalatal relationship related to the presence of crossbite in the anterior and buccal segments. Scoring using this system has an assessment from +1 to a score of −3 (Fig. 3). A score of +1 indicates more than a normal buccopalatal relationship, 0 indicates a normal buccopalatal relationship, −1 indicates an edge-to-edge relationship, and −2 indicates a moderate crossbite, with −3 indicating a severe crossbite (14,15,21).

**Figure 4:**
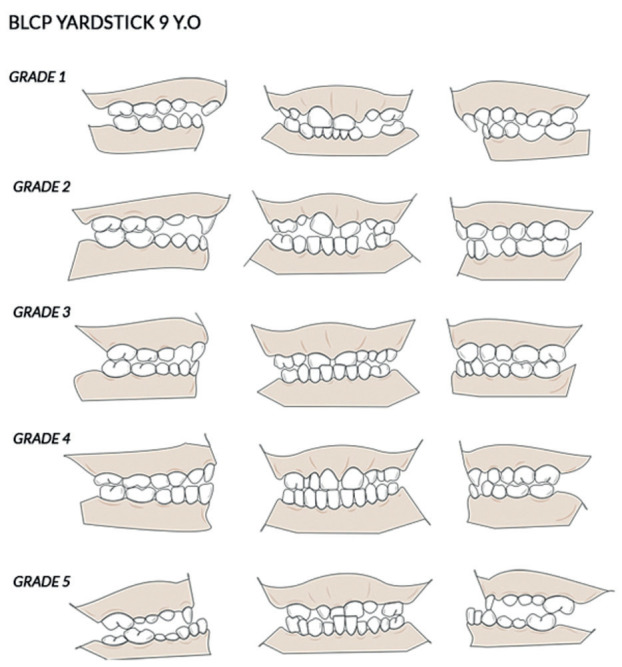
Illustration of the Bauru-BCLP yardstick study models for 9-year olds.

According to the Bauru-BCLP yardstick system, DAR assessment in the 9-year group was divided into five categories as follows: Score 1 = excellent result, score 2 = good result, score 3 = edge-to-edge apical base relationship, 4 = poor result, 5 = very poor result (Fig. 4). Scores 1 and 2 require only simple orthodontic treatment. A score of 3 requires more complex orthodontic treatment, while a score of 4, in addition to requiring complex orthodontic treatment, may require orthognathic surgical treatment. An absolute score of 5 requires orthognathic surgical treatment. DAR assessment in the 12-year group slightly differed from that for the 9-year group, where scores 1 and 2 were combined (12,13,14).

The correlation between the MHB index and Bauru-BCLP yardstick results determined using Spearman’s rank correlation coefficient for the 9-year and 12-year groups. The mean and median values for the two indicators were calculated from the mean measurements of each model, and each examiner made three measurements.

## Results

The study analysis included 32 subjects comprising 19 subjects in the 9-year group and 13 subjects in the 12-year group (Table 2). Study aims were explained to patient’s parents and signed informed consent was obtained. Clinical photos were then taken, and the maxillary and mandibular impressions were made and a record of the occlusion using wax material. Apparently, two patients could not undergo maxillary and mandibular impressions because their patients were uncooperative, and assessment was then taken using intraoral clinical photographs. The photographs cover both frontal and lateral aspects, including all the teeth involved in the measurement (central incisors, canines, and first molars on both sides of the maxilla).

-Average Kappa Value of 9-Year and 12-Year Age Groups Based on the MHB index and Bauru-BCLP yardstick

The number of evaluations of the incisor segment was taken from the combined number of assessments of the right and left incisors, while the number of evaluations of the lateral segment was taken from the combination of the assessments of the right and left canines and the right first molar and left first molar. According to the MHB index, in the incisor segment of the 9-year age group, 72 ratings were obtained from 3 examiners with an average Kappa value of 0.748, indicating that the examiner’s agreement level in this segment is satisfactory. In the lateral segment of the 9-year age group, 144 ratings were obtained from 3 examiners with an average Kappa value of 0.749, indicating that the examiner’s agreement level in this segment is also satisfactory. In the incisors segment of the 12-year-old group, 72 ratings were obtained from 3 examiners with an average Kappa value of 0.650, indicating that the examiner’s agreement level in this segment is satisfactory. In the lateral segment of the 12-year age group, 144 ratings obtained from 3 examiners with an average Kappa value of 0.640 indicate that the examiner’s agreement level in this segment is also satisfactory (Table 3).

**Table 3:**
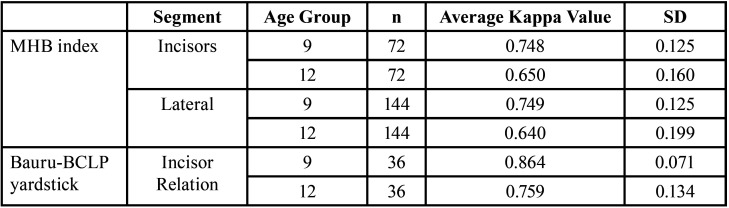
Average Kappa Value of 9- and 12-Year Age Groups Based on the MHB index and Bauru-BCLP yardstick.

According to the Bauru-BCLP yardstick, in the 9-year age group, 36 total assessments were obtained from 3 examiners with an average Kappa score of 0.864, indicating that the examiner’s agreement level is excellent. In the 12-year age group, 36 total assessments were also obtained from 3 examiners with an average Kappa score of 0.759, indicating that the examiner’s agreement level is satisfactory (Table 3).

-DAR Evaluation

Based on descriptive statistics on the Bauru-BCLP yardstick, the average DAR assessment in the 9-year age group was 2.92, with a range of interexaminer agreement values of 1.00–5.00. Furthermore, this average in the 12-year age group was 2.86, with an inter-examiner agreement range of 1.50–5.00 (Table 4).

**Table 4:**
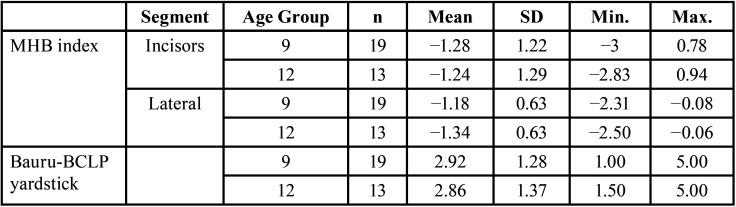
Descriptive Statistics of the MHB index and Bauru-BCLP yardstick Based on Average Value and Range of Inter-Rater Agreement Values.

-Descriptive Statistics of the MHB index Based on Average Value and Range of Inter-Examiner Agreement Values

The descriptive statistics of the MHB index showed the tendency of crossbite in both age groups. The average incisor segment assessment in the 9-year-old group was −1.28 (mild crossbite) in the interexaminer agreement range of −3–0.78, while in the lateral segment, it was −1.18 (mild crossbite) in the interexaminer agreement range of −2.31–−0.08. The results for the 12-year-old age group showed an average incisor segment assessment of −1.24 (mild crossbite) in the interexaminer agreement range of −2.83–0.94. In addition, the lateral segment was −1.34 (mild crossbite) in the interexaminer agreement range of −2.50–−0.06 (Table 4).

-Spearman Correlation Coefficients Between the MHB index and Bauru-BCLP yardstick

The obtained correlation coefficient for the 9-year age group in the incisor segment was −0.925, suggesting a very strong level of nonunidirectional relationship between the two indicators (Table 5). A statistically significant relationship existed between the MHB index and Bauru-BCLP yardstick incisor segment in the 9-year age group (Table 5). Similarly, the obtained correlation coefficient for the 12-year age group in the incisor segment was −0.934, indicating a very strong level of nonunidirectional relationship. A statistically significant relationship existed in the incisor segment between the MHB index and Bauru-BCLP yardstick in the 12-year age group (Table 5).

**Table 5:**
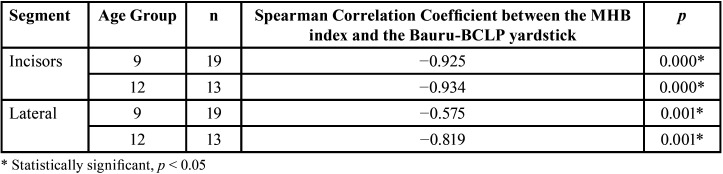
Spearman Correlation Coefficients Between the MHB index and Bauru-BCLP yardstick.

A moderate level of nonunidirectional relationship existed between the MHB index and Bauru-BCLP yardstick as the correlation coefficient for the 9-year age group in the lateral segment was −0.575 (Table 5). A statistically significant relationship existed on the lateral segment between the two indicators in the 9-year age group. In addition, a very strong and unidirectional relationship was observed between the two indicators as the correlation coefficient for the 12-year-old group in the lateral segment was −0.819. A statistically significant relationship existed in the lateral segment between the two indicators in the 12-year age group.

## Discussion

The evaluation of a cleft treatment includes speech function, DARs, maxillary growth, and aesthetic and psychosocial factors (1,4). DAR evaluation is crucial for assessing maxillofacial growth and is a key approach to assessing CLP treatment (9,10,11). The Bauru-BCLP yardstick and MHB index are reliable assessment measures of DARs in patients with BCLP (9,13,14). A good level of agreement was obtained from the inter-examiner assessment results for the 9-year-old group using the MHB index, both in the incisor and lateral segments (Kappa value range: 0.748–0.749). While the 9-year-old group had a good agreement level in the mild crossbite category, the 12-year age group based on the inter-examiner assessment of the MHB index also had a good agreement level in the incisor and lateral tooth segments (Kappa value range: 0.640–0.650) and was thus included in the mild crossbite category. A good agreement level between examiners using the MHB index, which was observed in this study, corroborated that of Tothill and Mossey’s study, in which 19 subjects with four examiners were considered, with a moderate-to-good agreement level observed between examiners (6). Mossey’s research was also used as a pilot study to modify the HB scoring system, having been modified by Heidbuchel and Kuijpers in 1997 from the initial concept of the Huddart–Bodenham system by Huddart and Bodenham in 1973 (6,15,21).

In both the 9- and 12-year-old groups in this study, the DAR assessment using the MHB index consistently resulted in a mild crossbite category, where the maxillary teeth were aligned with the mandibular teeth in a vertical direction (edge to edge), both in the anterior and lateral segments. This mild crossbite condition only requires more complex orthodontic treatment without orthognathic surgery (14,21).

An excellent level of interexaminer agreement (Kappa value 0.864) was observed while assessing subjects in the 9-year-old group using the Bauru-BCLP yardstick. The agreement level was in the mild deviation or edge-to-edge incisor relations (the Bauru-BCLP yardstick value was rounded to 3). In the 12-year-old group, the agreement between examiners was satisfactory (Kappa value 0.759). The satisfactory agreement in the 12-year-old group was in the minor deviation or edge-to-edge incisor relation category. This agreement results also corroborated the 2011 finding of Ozawa *et al*. who modified the GOSLON yardstick scoring system for patients with unilateral cleft lip. The research used 776 study models and involved 11 examiners from 5 countries, resulting in a good-to-very good agreement between examiners. Research from Ozawa and colleagues also produced five reference study models using the Bauru-BCLP yardstick scoring system (13). The results obtained also corroborated those of Batra and colleagues who examined 50 study models from Indian patients with three examiners in 2018. The research results had a very good agreement, with the surgery results, according to the Bauru-BCLP yardstick category, being good (the overall Bauru-BCLP yardstick score 2.36) (9).

In this study, measurements using the MHB index and Bauru-BCLP yardstick for the 9-year-old group in the lateral and incisor segments resulted in a moderate-to-very strong nonunidirectional relationship (correlation value: −0.575–−0.925). In the 12-year-old group, the agreement level between examiners using the two indicators resulted in a very strong nonunidirectional relationship (correlation value: −0.819–−0.934), both in the lateral and incisor segments. Bartzela *et al*. compared reliability levels between the two indicators and found the Spearman correlation coefficient to range from moderate nonunidirectional (−0.47) to very strong nonunidirectional relationship (−0.82). This Spearman correlation result explains the strong relationship between the two scoring systems (14,30).

The present study uncovers the DAR of patients with CBCLP after lip and palate repair at Harapan Kita Hospital, Jakarta, revealing mild crossbite or mild deviation category. The clinical condition of this mild crossbite or mild deviation describes an edge-to-edge tooth relationship. Patients with CBCLP tend to have severe malocclusion due to the extensive scarring of the palate region, particularly at the surgical site, resulting in medial traction of the palate and maxillary arch (28,29). Conditions with mild crossbite or mild deviation category after cleft surgery at Harapan Kita Hospital were made possible using the Manchester lip repair and modified pushback palate repair techniques as their protocol.

Bardach *et al*. found lip pressure after lip repair in rabbits (32). Indications exist for the relationship between lip pressure amount and anterior–posterior maxillary growth restriction after lip repair (32-35). The advantages of the Manchester lip repair technique include adequate lip length and lip conditions that are not too retracted horizontally (25,26). Furthermore, the palate repair using partial split lateral incision reduces the exposed bony areas in the lateral part of the hard palate. This approach results in faster epithelialization of the surgical wound compared to conventional techniques (full-thickness lateral incision). The rapid reepithelialization process reduces wound contraction and the potential for scar tissue to form, resulting in better maxillary growth. The amount of palatal scar tissue that occurs is directly related to the area of denuded palatal bone due to elevation of the mucoperiosteum during palate repair (27,28,29).

## Conclusions

The CLP repair protocol at Harapan Kita Children and Mother Hospital Jakarta is effective in the management of CBCLP cases with satisfactory results. The MHB index and Bauru-BCLP yardstick can be used to assess the DAR of patients with operated CBCLP.
